# Natural History of a Mouse Model Overexpressing the Dp71 Dystrophin Isoform

**DOI:** 10.3390/ijms222312617

**Published:** 2021-11-23

**Authors:** Kenji Rowel Q. Lim, Md Nur Ahad Shah, Stanley Woo, Harry Wilton-Clark, Pavel Zhabyeyev, Faqi Wang, Rika Maruyama, Gavin Y. Oudit, Toshifumi Yokota

**Affiliations:** 1 Department of Medical Genetics, Faculty of Medicine and Dentistry, University of Alberta, Edmonton, AB T6G2H7, Canada; kenjirow@ualberta.ca (K.R.Q.L.); mdnuraha@ualberta.ca (M.N.A.S.); skwoo@ualberta.ca (S.W.); hwiltonc@ualberta.ca (H.W.-C.); yokotama@ualberta.ca (R.M.); 2 Department of Medicine, Faculty of Medicine and Dentistry, University of Alberta, Edmonton, AB T6G2G3, Canada; zhabyeye@ualberta.ca (P.Z.); faqi@ualberta.ca (F.W.); 3 Mazankowski Alberta Heart Institute, University of Alberta, Edmonton, AB T6G2B7, Canada; 4 Muscular Dystrophy Canada Research Chair, Edmonton, AB T6G2H7, Canada

**Keywords:** dystrophin, Dp71, Duchenne muscular dystrophy, hDMDdel52 mice, del52, WT mice, cardiac dysfunction, dystrophic animal model

## Abstract

Dystrophin is a 427 kDa protein that stabilizes muscle cell membranes through interactions with the cytoskeleton and various membrane-associated proteins. Loss of dystrophin as in Duchenne muscular dystrophy (DMD) causes progressive skeletal muscle weakness and cardiac dysfunction. Multiple promoters along the dystrophin gene (*DMD*) give rise to a number of shorter isoforms. Of interest is Dp71, a 71 kDa isoform implicated in DMD pathology by various animal and patient studies. Strong evidence supporting such a role for Dp71, however, is lacking. Here, we use del52;WT mice to understand how Dp71 overexpression affects skeletal and cardiac muscle phenotypes. Apart from the mouse *Dmd* gene, del52;WT mice are heterozygous for a full-length, exon 52-deleted human *DMD* transgene expected to only permit Dp71 expression in muscle. Thus, del52;WT mice overexpress Dp71 through both the human and murine dystrophin genes. We observed elevated Dp71 protein in del52;WT mice, significantly higher than wild-type in the heart but not the tibialis anterior. Moreover, del52;WT mice had generally normal skeletal muscle but impaired cardiac function, exhibiting significant systolic dysfunction as early as 3 months. No histological abnormalities were found in the tibialis anterior and heart. Our results suggest that Dp71 overexpression may have more detrimental effects on the heart than on skeletal muscles, providing insight into the role of Dp71 in DMD pathogenesis.

## 1. Introduction

Dystrophin is a large, 427 kDa membrane-associated protein with a critical role in maintaining muscle membrane integrity. It has four main functional domains: an actin-binding domain (encoded by exons 1–8) at the N-terminus, a central rod domain (encoded by exons 8–64), a cysteine-rich domain (encoded by exons 64–70), and a C-terminal domain (encoded by exons 71–79) [1]. The specific function of each of these domains ultimately enables dystrophin to link the F-actin cytoskeleton in muscle fibers to the surrounding extracellular matrix, through a network of sarcolemmal glycoproteins (i.e., the dystrophin glycoprotein complex or DGC). This linkage provides mechanical reinforcement to the sarcolemma and protects it from stress during muscle contraction [2]. Mutations in the *DMD* gene that code for dystrophin lead to a group of disorders called the dystrophinopathies, of which the most prominent member is the fatal Duchenne muscular dystrophy (DMD). DMD is an X-linked recessive genetic disorder primarily characterized by progressive muscle degeneration and weakness [3]. About 1 in 3500–5000 males are born with DMD worldwide [4], and currently there is no cure for this disease apart from measures aimed at symptom management.

Expression of the full-length *DMD* transcript (Dp427) is independently regulated by three promoters. Each of these drives the expression of a version of Dp427 in different tissues, e.g., cortical neurons/hippocampus (B promoter) [5], cerebellar Purkinje cells/skeletal muscle (P promoter) [6], and skeletal/cardiac muscle (M promoter; primary full-length dystrophin) [5]. In addition to these Dp427 promoters, the *DMD* gene has at least four more internal promoters (at exons 30, 45, 56, and 63) that can produce shorter isoforms, each generating a dystrophin protein product named after their corresponding size, i.e., Dp260, Dp140, Dp116, and Dp71. Dp260 is expressed in the retina [7], Dp140 in the central nervous system and kidney [8], and Dp116 in Schwann cells [9]. Dp71 is expressed ubiquitously [10,11].

Even though the roles of full-length dystrophin have already been extensively studied, the physiological functions of its shorter isoforms are not well understood. Of particular interest would be Dp71, not only because it is expressed in muscle but also because previous works have implicated Dp71 in DMD pathogenesis [12,13]. Dp71 lacks both the N-terminal actin-binding and central rod domains of full-length dystrophin, rendering it unable to link muscle cell membranes to the cytoskeleton [14]. However, Dp71 retains the ability to bind proteins typically associated with the C-terminal end of Dp427, which could affect full-length dystrophin function. Indeed, one study showed that overexpression of human Dp71 in mice had a dominant negative effect on Dp427 and caused skeletal muscle degeneration [14]. In another study, Dp71 upregulation in the heart was associated with Purkinje fiber degeneration in a dystrophin-deficient dog model [13]. Clinical data from DMD patients revealed that mutations in the C-terminal domain, and in particular those affecting Dp71, were associated with decreased wheelchair use [15]. C-terminal dystrophin mutations were also associated with less severe cardiac symptoms in DMD patients [16,17]; however, there are studies that found no such correlations [18,19]. Overall, while Dp71 has been suggested to contribute to DMD pathology, such evidence has been scarce and not consistently supported. The physiological and pathological roles of Dp71 remain to be elucidated.

In this study, we aimed to examine the effects of Dp71 overexpression on skeletal and cardiac muscle phenotypes using a humanized, transgenic mouse model. This model contains a full-length, human *DMD* transgene on one copy of chromosome 5 with an out-of-frame partial deletion of exon 52. Due to the location of the deletion, only the expression of dystrophin isoforms with promoters downstream of exon 52 is permitted—in the case of muscle, this would be Dp71 [20,21,22]. Moreover, as this transgene is on a wild-type background (del52;WT), this leads to an overexpression model where the Dp71 protein comes from both human and murine sources. We also performed comparisons of skeletal and cardiac muscle phenotypes with the mdx mouse, an established dystrophin-deficient mouse model that harbors a nonsense mutation in the *DMD* gene [23]. Our natural history study found that del52;WT mice did not exhibit remarkable defects in skeletal muscle function and structure. A slight impairment of cardiac function was observed across age with corresponding structural alterations in the heart.

## 2. Results

### 2.1. del52;WT Mice Have Significantly Elevated Dp71 Levels in the Heart but Not Skeletal Muscle, and Have Mostly Normal Skeletal Muscle Function

**Figure 1:**
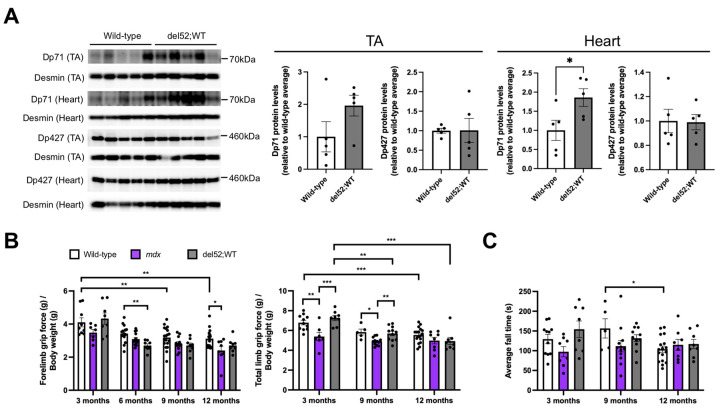
Dp71 overexpression and skeletal muscle function in del52;WT mice. (**A**) Western blotting with an antibody targeting the dystrophin C-terminal domain was done using total protein extracts from the tibialis anterior (TA) and the heart (12 μg each) of 6-month-old wild-type and del52;WT mice. Desmin was detected as a loading control. Quantification of Dp427 and Dp71 protein is on the right, with levels expressed relative to the average intensity of the corresponding wild-type samples for each tissue. Error bars: SEM, *n* = 5/group, * *p* < 0.05, unpaired two-tailed *t*-test. (**B**) Forelimb and total limb grip strength across age for wild-type, mdx, and del52;WT mice. Values are normalized to body weight. (**C**) Average fall times on the rotarod test across age. Dots represent individual mice. For (**B**,**C**), error bars: SEM, *n* = 5–19 (wild-type), 7–14 (mdx), 8–11 (del52;WT), * *p* < 0.05, ** *p* < 0.01, *** *p* < 0.001, one-way ANOVA with Tukey’s test.

Western blotting with an anti-C-terminal dystrophin antibody revealed that del52;WT mice had significantly increased levels of Dp71 protein in the heart (*p* < 0.05) compared to wild-type mice (Figure 1A). Dp71 levels appeared elevated in the tibialis anterior of del52;WT mice but were non-significant. On the other hand, Dp427 levels were similar between del52;WT and wild-type mice. To determine the effect of increased Dp71 levels on skeletal muscle function, grip strength and rotarod tests were performed on wild-type, mdx, and del52;WT mice at 3, 6, 9, and 12 months. Forelimb grip strength was significantly reduced in del52;WT compared to wild-type mice at 6 months (*p* < 0.01), but not at 3, 9, or 12 months (Figure 1B), while mdx mice displayed significantly reduced forelimb grip strength versus wild-type only at 12 months (*p* < 0.05). No other significant differences between groups were observed within each age. A significant decline in forelimb grip strength was observed for wild-type mice at later ages (*p* < 0.01), which was not observed for mdx or del52;WT mice. As for total limb grip strength, wild-type and del52;WT mice had similar performance at all ages examined (Figure 1B). At 3 and 9 months, mdx mice had significantly reduced total limb grip strength than either wild-type or del52;WT mice (at least *p* < 0.05), while del52;WT mice showed a significant decline of total limb grip strength with age (3 months vs. 9 months, *p* < 0.01; 3 months vs. 12 months, *p* < 0.001). Wild-type mice exhibited a similar behavior only between 3 and 12 months (*p* < 0.001). No significant differences in rotarod test performance were observed between the three groups of mice at all ages examined (Figure 1C).

### 2.2. Cardiac Function in del52;WT Mice Shows Signs of Impairment

**Figure 2:**
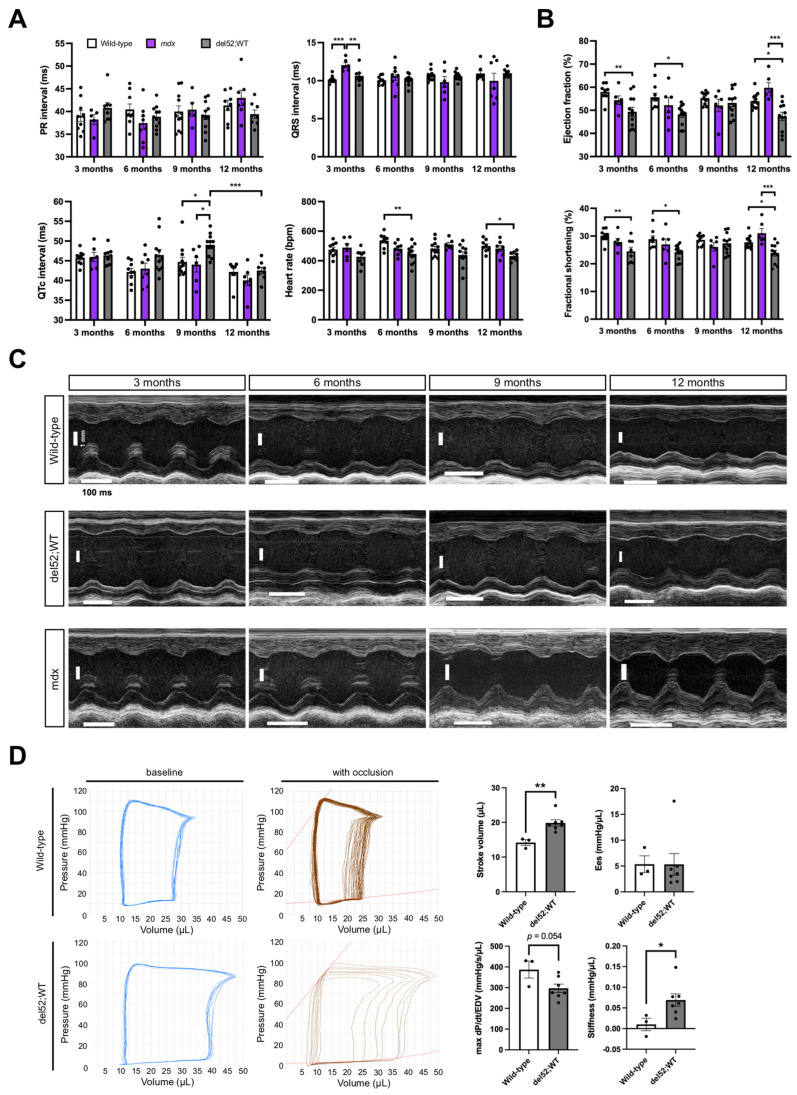
Cardiac function in del52;WT mice. (**A**) Electrocardiography results across age for wild-type, mdx, and del52;WT mice. (**B**) Ejection fraction and fractional shortening across age, as obtained through echocardiography. For (**A**,**B**), error bars: SEM, *n* = 8–12 (wild-type and del52;WT), 5–8 (mdx), * *p* < 0.05, ** *p* < 0.01, *** *p* < 0.001, one-way ANOVA with Tukey’s test. (**C**) Representative M-mode (parasternal long axis) echocardiography images of the left ventricle in the three groups of mice across age. (**D**) Representative pressure/volume loops at baseline and with occlusion for wild-type and del52;WT mice at 6 months are shown on the left. Quantification of selected parameters from the loops is shown on the right. Dots represent individual mice. Error bars: SEM, *n* = 3 (wild-type), 7 (del52;WT), * *p* < 0.05, ** *p* < 0.01, unpaired two-tailed *t*-test.

To determine the consequences of elevated Dp71 levels in the heart of del52;WT mice, we performed cardiac phenotyping on wild-type, mdx, and del52;WT mice through electrocardiography, echocardiography, and invasive pressure-volume analysis. PR and QRS intervals were mostly similar between genotypes across age (Figure 2A). The corrected QT interval was significantly prolonged in del52;WT compared to wild-type or mdx mice at 9 months (*p* < 0.05) but was otherwise similar in other ages. While del52;WT mice also exhibited significantly slower heart rates than wild-type mice at 6 (*p* < 0.01) and 12 months (*p* < 0.05), mdx mice showed no detectible differences from wild-type mice in all parameters, save an increased QRS interval at 3 months (*p* < 0.001).

In terms of echocardiographic parameters, del52;WT mice had significantly reduced left ventricle (LV) ejection fraction and fractional shortening than wild-type mice at 3, 6, and 12 months (at least *p* < 0.05) or mdx mice at 12 months (*p* < 0.001) (Figure 2B); mdx mice had significantly increased LV ejection fraction and fractional shortening than wild-type mice at 12 months (*p* < 0.05). Cardiac structure was altered in del52;WT mice, which was especially evident at 3 months (Figure 2C). In particular, del52;WT mice had significantly thinner LV posterior walls than wild-type mice in at least one phase of the cardiac cycle (systole/diastole) across all ages examined (at least *p* < 0.05); this was also the case when compared with mdx mice at 6 months of age onward (at least *p* < 0.05) (Table 1). At 12 months, del52;WT mice had significantly increased systolic LV volumes versus wild-type mice (*p* < 0.01). Left atrium (LA) diameters were likewise significantly increased in del52;WT than wild-type mice, which was consistently observed up to 9 months of age (3, 6 months, *p* < 0.01; 9 months, *p* < 0.05). On the other hand, mdx mice showed significant LA dilation at 3 (*p* < 0.05) and 6 months (*p* < 0.01), as well as systolic LV posterior wall thinning at 6 months (*p* < 0.05) compared to wild-type mice. Moreover, mdx mice had decreased LV internal diameters and volumes (diastolic/systolic) at 9 and 12 months compared to both wild-type and del52;WT mice (*p* < 0.01). Differences in other echocardiographic parameters, e.g., those pertaining to the mitral valve, were either absent or inconsistent between the three groups of mice across age.

Pressure–volume measurements at 6 months revealed that the pressure-volume loops of del52;WT mice were wider than those of wild-type mice (Figure 2D). Indeed, stroke volume was significantly elevated in del52;WT mice at this age (*p* < 0.01). Maximum dP/dt/EDV values were visibly reduced in del52;WT mice, but not statistically significant (*p* = 0.054). Evaluation of end-systolic and end-diastolic pressure–volume relationships through occlusion of the inferior vena cava showed that del52;WT hearts had no notable changes in contractility but had significantly stiffer LVs (*p* < 0.05), respectively.

### 2.3. del52;WT Mice Have Histologically Normal Skeletal and Cardiac Muscle

**Figure 3:**
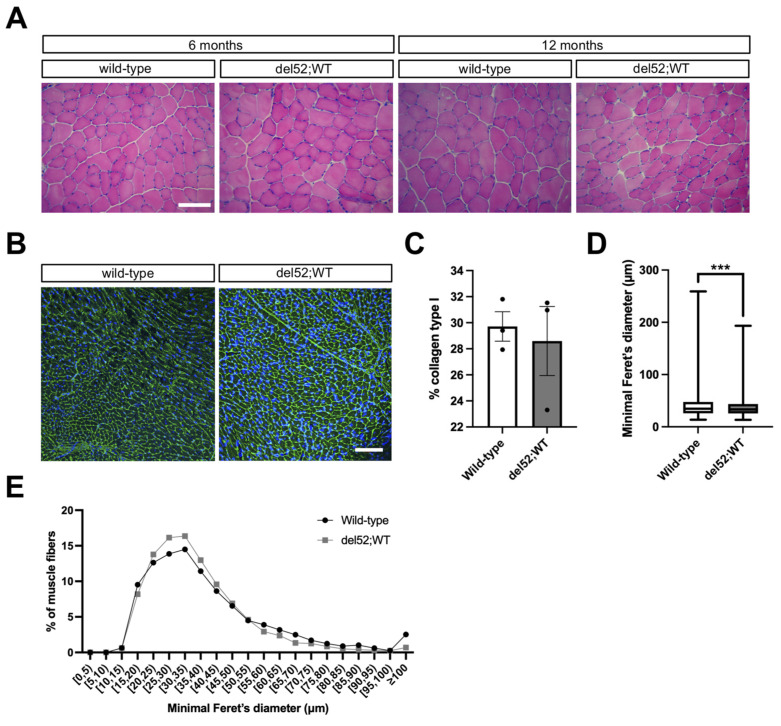
Histological analysis of skeletal and cardiac muscle in del52;WT mice. (**A**) Representative hematoxylin/eosin-stained images of tibialis anterior muscles in wild-type and del52;WT mice at 6 and 12 months. Scale bar: 100 μm. (**B**) Representative immunofluorescence images of collagen type I (green) and nuclei (blue) in the cardiac muscle of wild-type and del52;WT mice at 12 months. Scale bar: 100 μm. (**C**) Quantification of collagen type I area in cardiac muscle immunofluorescence images. Dots represent individual mice. Error bars: SEM, *n* = 3/group. (**D**) Quantification of the minimal Feret’s diameter of individual cardiomyocytes in cardiac muscle immunofluorescence images. Box: P_25_-P_75_, central line: median, whiskers: range. *n* = 3/group (average 1535 and 1951 fibers counted per replicate for wild-type and del52;WT, respectively), *** *p* < 0.001, unpaired two-tailed *t*-test. (**E**) Data in (**D**), but as a frequency distribution.

Finally, we stained tibialis anterior sections from wild-type and del52;WT mice with hematoxylin/eosin (HE) to assess the effects of Dp71 overexpression on overall skeletal muscle structure. For cardiac tissue sections, we performed immunofluorescent staining for collagen type I to study effects on fibrosis and cardiomyocyte size, two cellular phenotypes commonly affected in the dystrophic heart [24,25]. We did not notice any major differences between del52;WT and wild-type mice in these histological tests. HE staining revealed that del52;WT tibialis anterior muscles were histologically normal and indistinguishable from wild-type in terms of the absence of dystrophic landmarks such as fibrosis/necrosis, inflammatory infiltrates, and centrally-nucleated fibers (Figure 3A). Collagen staining was also similar between the two mice (Figure 3B), with no differences in the collagen type I-positive areas observed (Figure 3C). Measuring the minimal Feret’s diameter of individual cardiomyocytes in collagen type I-stained sections showed that del52;WT hearts had slightly smaller cardiomyocytes than those of wild-type mice, a difference that was statistically significant (*p* < 0.001) (Figure 3D,E).

## 3. Discussion

We evaluated the effects of Dp71 overexpression on skeletal and cardiac muscle phenotypes in del52;WT mice. This model relies entirely on endogenous expression from a full-length, exon 52-deleted human *DMD* transgene to achieve Dp71 overexpression. We saw that the Dp71 protein levels observed in del52;WT mice were modestly elevated compared to those in other transgenic mice, which carried Dp71 constructs under the control of dedicated promoters [26]. Dp71 protein levels in previous models were qualitatively at least 3-fold higher than those in wild-type mice in the skeletal muscle and heart based on Western blot images [14], as opposed to our del52;WT mice, which showed only 1.2- and 1.3-fold higher levels in these tissues, respectively (Figure 1A). This contrast in Dp71 overexpression likely explains the different natural history of our model, or why we failed to observe any dominant negative effect of Dp71 on full-length dystrophin in skeletal muscle (Figure 1A) as had been shown previously [14].

The role of Dp71 in differentiated skeletal muscle remains uncertain, due in part to many studies demonstrating its absence in this tissue [10,11,27]. However, we successfully detected Dp71 protein in both wild-type and del52;WT adult skeletal muscle (Figure 1A). Using a sensitive capillary Western assay system, another group was also able to detect Dp71 protein in healthy human and murine skeletal muscle [11]. Further work is therefore required to confirm the presence of Dp71 in skeletal muscle. On a different note, we did not find any strong impairment in the skeletal muscle structure or function of del52;WT mice, i.e., the tibialis anterior muscles of del52;WT mice were histologically normal (Figure 3A), and their grip strength was mostly similar to that of wild-type controls (Figure 1B). While our findings agree with previous work that showed a lack of skeletal muscle dystrophy in another Dp71-overexpressing mouse model [28], it should be considered that we did not achieve robust levels of Dp71 overexpression in del52;WT skeletal muscle (Figure 1A). Interestingly, when Dp71 was overexpressed in a dystrophic mdx and not a wild-type mouse background by other groups, skeletal muscle histopathology was exacerbated [12,14,28]. With the observation that Dp71 expression is naturally decreased in the course of myogenesis [29,30], it is possible that the physiological or pathological contributions of Dp71 in skeletal muscle are better appreciated in an environment where there is no full-length dystrophin or where the level of full-length dystrophin present is below a certain threshold. Notably, in these previous studies, it was also found that Dp71 overexpression in mdx mice restored the localization of DGC proteins to the sarcolemma [12,28]. The reason why DGC restoration by Dp71 did not ameliorate the dystrophic pathology in mdx mice remains to be investigated. However, it has been suggested that the incomplete link between the DGC and cytoskeleton caused by the absence of the dystrophin N-terminal domain in Dp71 renders muscle cell membranes fragile, or that Dp71 may be orchestrating other, unknown functions through the DGC that would otherwise not be observed when Dp427 is present [12,28]. 

On the other hand, the effect of Dp71 overexpression was more pronounced in the heart, with del52;WT mice showing significant systolic dysfunction and pathological remodeling of the myocardium across most ages examined (Figure 2B–D, Table 1). Such phenotypes are reminiscent of the dilated cardiomyopathy found in DMD patients [31]. It is unknown if similar defects exist in other Dp71-overexpressing mouse models, as in-depth cardiac phenotype analysis was not performed in previous studies [12,14,28]. We observed a significant decrease in ejection fraction starting at 3 months of age (Figure 2B), strikingly earlier than that seen in mdx mice, where cardiac failure begins presenting at advanced ages (> 1 year) [32]. Despite these clear indications of cardiac pathology, we did not find any abnormal histological phenotypes (fibrosis, cardiomyocyte size) in the hearts of del52;WT mice (Figure 3B–E). Previous groups likewise failed to detect dystrophic histological phenotypes in hearts from transgenic Dp71-overexpressing mice on either wild-type or mdx backgrounds [14,28]. While we cannot exclude the possibility that Dp71 overexpression may have effects on the heart not captured by our simple histological assessment, our findings and those of others suggest that Dp71 may contribute towards the dystrophic cardiac phenotype more via effects on cellular function rather than structure. Moreover, our study (Figure 1A) and a previous report argue against a dominant negative mechanism of action against Dp427 in the heart [14], indicating that Dp71 may be driving pathology in cardiomyocytes through means other than disrupting the DGC. As the effects of Dp71 overexpression on skeletal muscle phenotypes also varied depending on disease background (wild-type versus mdx) [28], examining how Dp71 overexpression affects cardiac function in a dystrophic model may prove informative.

In summary, our assessment of the del52;WT model revealed that Dp71 overexpression was generally more detrimental to the heart than the skeletal muscle, at least in the context of a wild-type background. It would be interesting to perform a similar longitudinal natural history study on other Dp71 transgenic mouse models, particularly those having a stronger overexpression of Dp71 [12,14,28], to see whether the same phenotypes could be observed. Studying Dp71-deficient mice would also be helpful [33], as use of this model has recently led to valuable insights about the function of Dp71 in other tissues, such as the brain and retina [34,35]. Ultimately, the present work sets the stage for further investigating the cardiac pathology observed in del52;WT mice, as well as the role Dp71 has in driving this phenotype and its potential implications on the progression of DMD seen in patients.

## 4. Materials and Methods

### 4.1. Animals

Wild-type, mdx, and del52;WT mice on the C57BL6/J background were housed and cared for by the Health Sciences Laboratory Animal Services, University of Alberta. All animal experiments were approved by the Animal Care and Use Committee, University of Alberta Research Ethics Office (AUP00000365). Genotypes were confirmed by PCR [20,21,22]; only male mice that were heterozygous for the human exon 52-deleted *DMD* transgene were used for analysis. Mice received standard food and water ad libitum.

### 4.2. Muscle Function Tests

All skeletal muscle function tests were performed in the morning to minimize variability. Forelimb and total limb grip tests were performed blinded according to a standard protocol (TREAT-NMD SOP DMD_M.2.2.001). Grip strength was measured from at least three trials per mouse using a Chatillon DFE II meter (Columbus Instruments, Columbus, OH, USA), with the three most consistent readings averaged and divided by mouse body weight. Rotarod tests were performed blinded based on the protocol of Aartsma-Rus and van Putten (2014) [36], using the AccuRotor 4-channel rotarod (Omnitech Electronics, Columbus, OH, USA) and an acceleration profile of 5 to 45 rpm over 300 s. The average fall time from three trials spaced 15 min intervals apart was recorded for analysis. 

### 4.3. Electrocardiographic (ECG) Recording

Mice were placed under isoflurane anesthesia (1.5–2%) on a heated pad (body temperature maintained at 37 °C, measured by the rectal probe). ECG leads were placed in Lead I configuration. The signal was digitized using acquisition interface ACQ-7700 (Data Science International, St. Paul, MN, USA) with P3 Plus software (ver. 5.0, Data Science International, St. Paul, MN, USA) [37]. QT intervals were corrected for heart rate using the Bazett formula. 

### 4.4. Echocardiographic Imaging

Transthoracic M-mode and Doppler echocardiographic examinations were performed using the Vevo 3100 with a 40 MHz transducer (MX550S; VisualSonics, Toronto, Canada) as previously described [38]. Mice were placed on a heating pad and a nose cone with 2% isoflurane in 100% oxygen was applied. The temperature was maintained at 36.5 to 37.5 °C. Ultrasound gel was placed on the chest of the anesthetized mouse. The ultrasound probe was placed in contact with the ultrasound gel and scanning was performed over 30 min. The temperature and heart rate were constantly monitored during the scanning. M-mode images were used to measure LV chamber size and wall thickness, among other parameters.

### 4.5. Pressure-Volume Loop Measurements

To measure the LV pressure–volume relationship, a 1.2 F admittance catheter (Scisense Inc., London, ON, Canada) was used as previously described [38]. Briefly, mice were anesthetized with isoflurane, and an incision was made in the right carotid artery. A catheter was inserted into the incision and was advanced to the LV through the ascending aorta and aortic valve. The position of the catheter was monitored by pressure along with the magnitude and phase using the ADVantage pressure–volume system (Scisense Inc.) and iWorx data acquisition system (iWorx Systems, Inc., Dover, NH, USA) connected to the catheter. Once the desired range for magnitude and phase was achieved, a baseline scan was performed to derive volume using Baan’s equation, and the pressure–volume loop was recorded using LabScribe2 software (ver. 2.347, iWorx Systems, Inc.). The inferior vena cava was briefly occluded to obtain alterations in venous returns to derive end-systolic and end-diastolic pressure–volume relationships. The pressure–volume loops were analyzed using LabScribe2 software.

### 4.6. Western Blot

After functional testing, mice were euthanized by cervical dislocation and dissected. Freshly harvested tibialis anterior and heart samples were mounted on corks using tragacanth gum and snap-frozen in liquid nitrogen-cooled isopentane. Sections from these samples were prepared (20 μm) in cool 1.5 mL tubes, and protein was extracted using a complete protease inhibitor cocktail (Roche)-supplemented lysis buffer (70 mM Tris-HCl (pH 6.7), 5 mM EDTA (pH 8.0), 10% SDS, 5% β-mercaptoethanol in water) [39]. Proteins were quantified using Pierce^TM^ Coomassie (Bradford) (Thermo Fisher, Waltham, MA, USA) and diluted using the lysis buffer. Proteins were incubated with 1× LDS Sample Buffer and Reducing Agent (NuPAGE^TM^, Invitrogen, Waltham, MA, USA) at 70 °C for 10 min, and then loaded (12 μg) and run on a 3–8% tris-acetate gel (NuPAGE^TM^, Invitrogen) at 150 V for 75 min. A semi-dry transfer of proteins to a PVDF membrane (Millipore, Burlington, MA, USA) was performed at 20 V for 70 min, after which the membrane was blocked overnight in 2% ECL Prime Blocking agent (GE Healthcare, Chicago, IL, USA) at 4 °C. The following day, membranes were cut and incubated in either 1:2500 anti-dystrophin (ab15277, Abcam, Cambridge, UK) or 1:4000 anti-desmin antibody (ab8592, Abcam) in the blocking agent for 1 hr at room temperature. Membranes were then washed thrice with PBS containing 0.05% Tween 20 (PBST) for 10 min each at room temperature, before being incubated in 1:10,000 horseradish peroxidase-conjugated anti-rabbit IgG (H+L) antibody in PBST. Membranes were once again washed with PBST and incubated in prepared ECL Select Detection Reagent (GE Healthcare) for 5 min prior to visualization. Western blot quantification was done using ImageLab^TM^ Software, v.6.0.1 (Bio-Rad, Hercules, CA, USA). Dp427 and Dp71 band intensities were normalized to those of desmin and were expressed relative to the average protein levels observed in the wild-type mice.

### 4.7. Histology

Frozen skeletal and cardiac muscle sections (7 μm, on poly-L-lysine slides) were thawed at room temperature for at least 30 min prior to staining. HE staining was performed following standard procedure, using Mayer’s hematoxylin and eosin Y (Electron Microscopy Sciences), and mounted with Permount^TM^ (Fisher Scientific, Hampton, NH, USA). For immunofluorescence, sections were first blocked with 10% goat serum in phosphate buffered saline with 0.1% Triton X-100 (PBSTX) for 20 min at room temperature, followed by incubation with 1:50 of the anti-collagen type I antibody (ab34710, Abcam) in blocking agent for 1 hr at room temperature. Slides were then washed with PBS thrice for 5 min each, and then incubated in 1:2000 of Alexa Fluor 488-conjugated goat anti-rabbit IgG (H+L) antibody (Invitrogen) in PBSTX for 1 hr at room temperature. Slides were washed again with three 5-min incubations in PBS, and finally mounted with VECTASHIELD HardSet Antifade Mounting Medium with DAPI (Vector Laboratories, Burlingame, CA, USA). Blinded personnel visualized the samples at 200× magnification (Optika B-290TB for HE, Zeiss LSM 710 for immunofluorescence), taking images from three randomly selected fields of view for analysis. Quantification of immunofluorescent images was done using ImageJ (NIH). Collagen type I areas were measured by first applying a threshold (Huang), and then dividing by the area of the field of view. Cardiomyocyte minimal Feret’s diameters were semi-automatically quantified using a macro based on Open-CSAM [40]. 

### 4.8. Statistical Analysis

All statistical tests were performed using GraphPad Prism (ver. 9.0.1, GraphPad Software, San Diego, CA, USA). One-way ANOVA with post-hoc Tukey’s test or unpaired two-tailed *t*-test were conducted as appropriate, with a *p*-value less than at least 0.05 considered statistically significant.

## Figures and Tables

**Table 1:** Summary of echocardiographic parameters in wild-type, mdx, and del52;WT mice across age.

Parameter	3 Months			6 Months			9 Months			12 Months		
	WT	mdx	del52;WT	WT	mdx	del52;WT	WT	mdx	del52;WT	WT	mdx	del52;WT
Number	8	4–5	12	8	5–6	11	10	3–6	11–12	12	6	10–11
Heart rate	443.0 (31.3)	425.3 (32.8)	426.6 (48.7)	464.6 (32.7)	450.5 (50.1)	418.4 (36.8) *^b^*	423.8 (45.6)	528.7 (23.9) *^aa^*	430.4 (38.9) *^cc^*	427.1 (62.6)	531.5 (63.4) *^aa^*	393.3 (55.0) *^ccc^*
LVIDd	3.9 (0.2)	4.1 (0.2)	4.2 (0.4)	4.3 (0.4)	4.0 (0.4)	4.3 (0.3)	4.3 (0.3)	3.3 (0.4) *^aa^*	4.5 (0.4) *^cc^*	4.3 (0.2)	3.5 (0.5) *^aa^*	4.6 (0.4) *^cc^*
LVIDs	2.8 (0.2)	3.0 (0.2)	3.2 (0.5)	3.0 (0.3)	2.9 (0.4)	3.3 (0.3)	3.1 (0.2)	2.4 (0.3) *^aa^*	3.3 (0.4) *^cc^*	3.1 (0.2)	2.4 (0.3) *^aa^*	3.6 (0.4) *^cc^*
LVPWd	0.6 (0.1)	0.5 (0.2)	0.5 (0.1) *^bb^*	0.7 (0.05)	0.7 (0.1)	0.6 (0.1) *^bb,cc^*	0.7 (0.1)	0.8 (0.2)	0.5 (0.1) *^b,cc^*	0.6 (0.1)	0.7 (0.1)	0.5 (0.1) *^c^*
LVPWs	1.0 (0.1)	0.8 (0.2)	0.7 (0.1)	1.1 (0.1)	0.9 (0.2) *^a^*	0.8 (0.1) *^bb,c^*	1.0 (0.1)	1.1 (0.1)	0.8 (0.1) *^bb,cc^*	1.0 (0.2)	1.1 (0.2)	0.7 (0.2) *^bb,cc^*
LV vol s	29.1 (4.1)	34.1 (5.6)	41.2 (15.4)	36.3 (8.0)	33.6 (12.1)	44.1 (9.9)	37.7 (4.6)	20.9 (6.2) *^aa^*	43.8 (11.7) *^cc^*	38.4 (4.9)	20.1 (7.2) *^aa^*	54.0 (16.8) *^bb,cc^*
LV vol d	68.1 (8.1)	75.7 (7.1)	79.8 (19.4)	82.0 (16.4)	68.9 (15.2)	84.4 (15.6)	84.7 (12.4)	46.3 (12.9) *^aa^*	92.2 (17.7) *^cc^*	84.7 (7.0)	51.6 (18.9) *^aa^*	98.3 (18.4) *^cc^*
EF	57.9 (4.0)	54.3 (5.1)	49.3 (7.2) *^bb^*	55.7 (5.4)	52.8 (7.6)	48.2 (4.9) *^bb^*	55.3 (3.4)	52.1 (8.4)	53.2 (5.9)	53.9 (4.1)	59.8 (5.7) *^a^*	47.4 (7.1) *^bb,cc^*
FS	30.1 (2.7)	27.9 (3.3)	24.8 (4.4) *^bb^*	28.9 (3.7)	27.0 (4.8)	24.2 (2.9) *^bb^*	28.6 (2.3)	26.1 (4.8)	27.4 (3.7)	27.7 (2.7)	31.0 (4.2) *^a^*	23.8 (4.2) *^bb,cc^*
LA	1.5 (52.4)	1.6 (0.2) *^a^*	1.6 (0.04) *^bb^*	1.4 (0.1)	1.7 (0.3) *^aa^*	1.7 (0.1) *^bb^*	1.4 (0.1)	1.8 (0.7)	1.9 (0.3) *^b^*	1.6 (0.3)	1.9 (0.5)	1.9 (0.2)
IVRT	16.9 (2.0)	14.7 (2.1)	16.5 (4.2)	13.5 (1.0)	14.1 (1.4)	14.3 (1.8)	13.4 (1.6)	13.7 (2.4)	13.3 (1.5)	13.9 (2.0)	12.6 (5.0)	16.0 (2.7)
IVCT	16.7 (56.6)	13.9 (5.4)	16.1 (5.5)	13.5 (3.2)	10.9 (2.7)	12.5 (2.6)	11.7 (3.3)	10.5 (2.7)	10.9 (2.9)	12.3 (3.5)	9.5 (2.4)	13.8 (2.4) *^c^*
MV decel	22.4 (4.9)	18.3 (2.4)	22.0 (5.6)	17.7 (3.0)	16.9 (5.4)	24.5 (5.2) *^b,c^*	19.8 (6.0)	14.5 (7.2)	22.9 (5.6)	16.2 (4.6)	16.5 (7.9)	19.8 (3.5)
MV E	663.5 (85.5)	702.6 (70.6)	681.9 (118.6)	619.5 (65.7)	713.8 (118.2)	697.8 (76.1)	610.8 (46.7)	476.8 (196.5)	727.9 (114.4) *^cc^*	610.5 (36.8)	541.5 (206.2)	636.4 (103.0)
MV A	476.0 (43.7)	444.2 (65.1)	438.5 (99.6)	437.9 (92.8)	513.8 (102.9)	452.3 (98.1)	423.2 (66.5)	368.7 (179.0)	458.2 (119.9)	441.6 (102.3)	412.9 (151.1)	420.2 (178.2)
MV E/A	1.4 (0.2)	1.6 (0.2)	1.6 (0.3)	1.5 (0.2)	1.4 (0.2)	1.6 (0.4)	1.5 (0.2)	1.6 (1.2)	1.7 (0.4)	1.4 (0.3)	1.6 (1.0)	1.7 (0.4)
E’	−24.6 (4.6)	−19.0 (2.8)	−23.6 (3.9)	−23.5 (5.5)	−25.8 (3.6)	−22.7 (3.3)	−23.1 (4.2)	−27.4 (4.5)	−21.8 (3.6) *^c^*	−19.1 (4.0)	−24.3 (9.5)	−18.0 (5.2)
A’	−24.3 (5.3)	−26.9 (5.1)	−23.2 (1.8)	−22.9 (4.8)	−32.1 (9.3) *^a^*	−21.7 (3.6) *^cc^*	−21.1 (5.4)	−26.3 (5.2)	−23.8 (3.2)	−23.5 (5.0)	−27.5 (11.8)	−22.0 (6.9)
E’/A’	1.0 (0.2)	0.7 (0.04) *^a^*	1.0 (0.2) *^c^*	1.1 (0.3)	0.9 (0.3)	1.1 (0.3)	1.2 (0.4)	1.1 (0.3)	0.9 (0.2)	0.9 (0.3)	1.0 (0.7)	0.9 (0.3)
MV E/E’	−28.2 (8.8)	−37.5 (6.8)	−29.6 (6.7)	−27.9 (7.9)	−29.0 (6.4)	−31.3 (5.9)	−27.2 (5.3)	−17.5 (8.7)	−34.1 (6.3) *^b,cc^*	−33.1 (6.1)	−26.8 (16.0)	−38.1 (12.5)

*a*—WT vs. mdx; *b*—WT vs. del52;WT; *c*—mdx vs. del52;WT; number of letters indicate significance, i.e., for a letter *n*, *^n^ p* < 0.05, *^nn^ p* < 0.01, *^nnn^ p* < 0.001 (one-way ANOVA, post-hoc Tukey’s). Abbreviations: LVID—left ventricular interior diameter; LVPW—left ventricle posterior wall; EF—ejection fraction; FS—fractional shortening; LA—left atrium; IVRT—isovolumic relaxation time; IVCT—isovolumic contraction time; MV—mitral valve; d—diastole; s—systole, WT—wild-type.

## Data Availability

All data are available in the manuscript.
